# Crystal structure of benzyl 3-(3-methyl­phen­yl)di­thio­carbazate

**DOI:** 10.1107/S2056989015004764

**Published:** 2015-03-14

**Authors:** NurFadhilah Abdul Aziz, Enis Nadia Md Yusof, Thahira Begum S. A. Ravoof, Edward R. T. Tiekink

**Affiliations:** a Department of Chemistry, Universiti Putra Malaysia, 43400 Serdang, Malaysia; b Department of Chemistry, University of Malaya, 50603 Kuala Lumpur, Malaysia

**Keywords:** crystal structure, hydrogen bonding, C—H⋯π inter­actions, *S*-substituted di­thio­carbazate

## Abstract

In the title compound, C_15_H_16_N_2_S_2_, the central CN_2_S_2_ residue is almost planar (r.m.s. deviation = 0.0354 Å) and forms dihedral angles of 56.02 (4) and 75.52 (4)° with the phenyl and tolyl rings, respectively; the dihedral angle between the aromatic rings is 81.72 (5)°. The conformation about the N—N bond is *gauche* [C—N—N—C = −117.48 (15)°]. Overall, the mol­ecule has the shape of the letter *L*. In the crystal packing, supra­molecular chains along the *a* axis are formed by N—H⋯S(thione) hydrogen bonds whereby the thione S atom accepts two such bonds. The hydrogen bonding leads to alternating edge-shared eight-membered {⋯HNCS}_2_ and 10-membered {⋯HNNH⋯S}_2_ synthons. The chains are connected into layers by phen­yl–tolyl C—H⋯π inter­actions; the layers stack along the *c* axis with no specific inter­actions between them.

## Related literature   

For background on the coordination chemistry of di­thio­carbazate derivatives, see: Ravoof *et al.* (2010 ▸). For the structure of the 2-tolyl analogue, which is superimposable upon the title compound with the exception of the tolyl rings, see: Tayamon *et al.* (2012 ▸). For the synthesis, see: Tarafder *et al.* (2002 ▸).
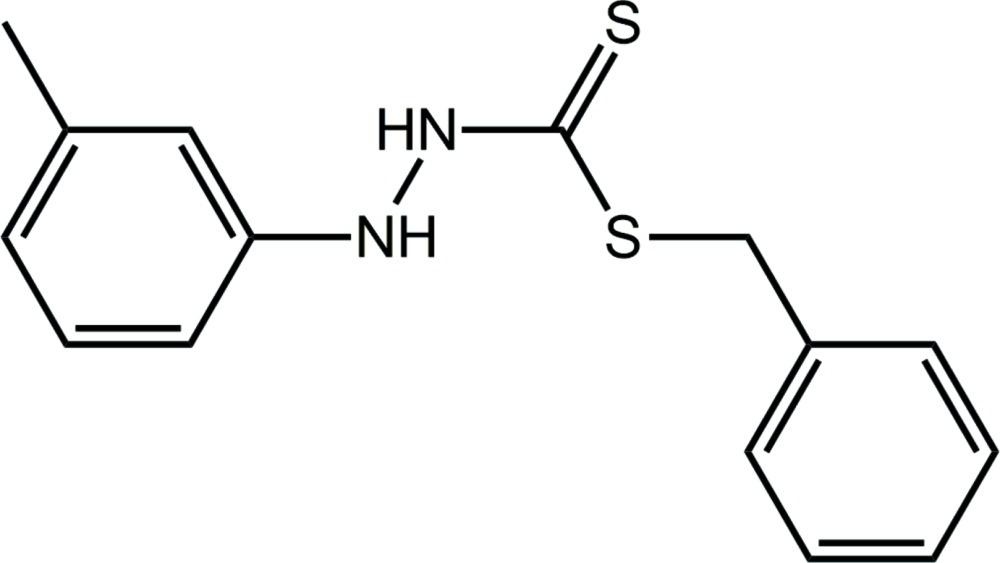


## Experimental   

### Crystal data  

C_15_H_16_N_2_S_2_
*M* *_r_* = 288.42
Monoclinic, 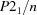

*a* = 5.9396 (1) Å
*b* = 10.3243 (2) Å
*c* = 23.5474 (5) Å
β = 96.952 (2)°
*V* = 1433.36 (5) Å^3^
*Z* = 4
Cu *K*α radiation
μ = 3.25 mm^−1^
*T* = 100 K
0.20 × 0.09 × 0.06 mm

### Data collection  

Oxford Diffraction Xcaliber Eos Gemini diffractometer
Absorption correction: multi-scan (*CrysAlis PRO*; Agilent, 2011 ▸) *T*_min_ = 0.700, *T*_max_ = 1.000
18486 measured reflections
2784 independent reflections
2616 reflections with *I* > 2σ(*I*)
*R*_int_ = 0.022

### Refinement  

*R*[*F* ^2^ > 2σ(*F* ^2^)] = 0.036
*wR*(*F* ^2^) = 0.104
*S* = 1.01
2784 reflections
179 parameters
2 restraints
H atoms treated by a mixture of independent and constrained refinement
Δρ_max_ = 0.43 e Å^−3^
Δρ_min_ = −0.31 e Å^−3^

### 

Data collection: *CrysAlis PRO* (Agilent, 2011 ▸); cell refinement: *CrysAlis PRO*; data reduction: *CrysAlis PRO*; program(s) used to solve structure: *SHELXS97* (Sheldrick, 2015 ▸); program(s) used to refine structure: *SHELXL2014* (Sheldrick, 2015 ▸); molecular graphics: *ORTEP-3 for Windows* (Farrugia, 2012 ▸), *QMOL* (Gans & Shalloway, 2001 ▸) and *DIAMOND* (Brandenburg, 2006 ▸); software used to prepare material for publication: *publCIF* (Westrip, 2010 ▸).

## Supplementary Material

Crystal structure: contains datablock(s) 1, I. DOI: 10.1107/S2056989015004764/hb7378sup1.cif

Structure factors: contains datablock(s) I. DOI: 10.1107/S2056989015004764/hb7378Isup2.hkl

Supporting information file. DOI: 10.1107/S2056989015004764/hb7378Isup3.cml

**Fig. 1.**
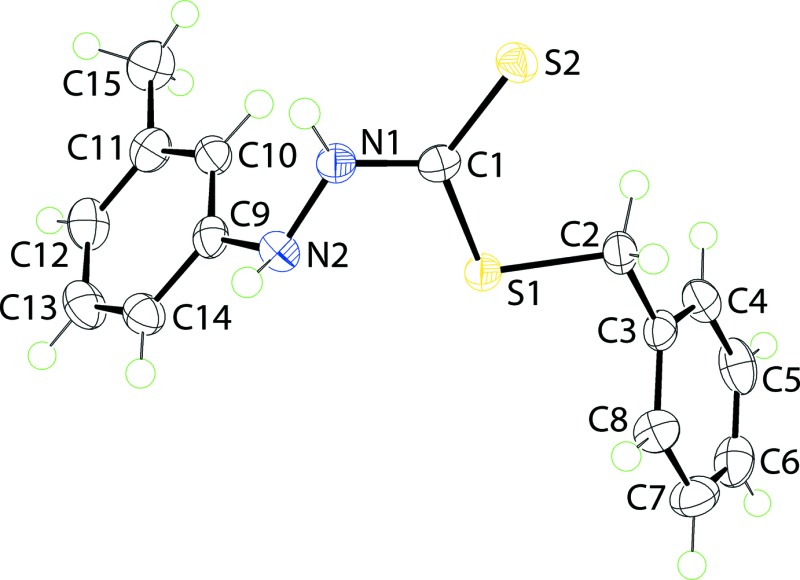
The molecular structure of the title compound showing displacement ellipsoids at the 70% probability level.

. DOI: 10.1107/S2056989015004764/hb7378fig1.tif
The mol­ecular structure of the title compound showing displacement ellipsoids at the 70% probability level.

**Fig. 2.**
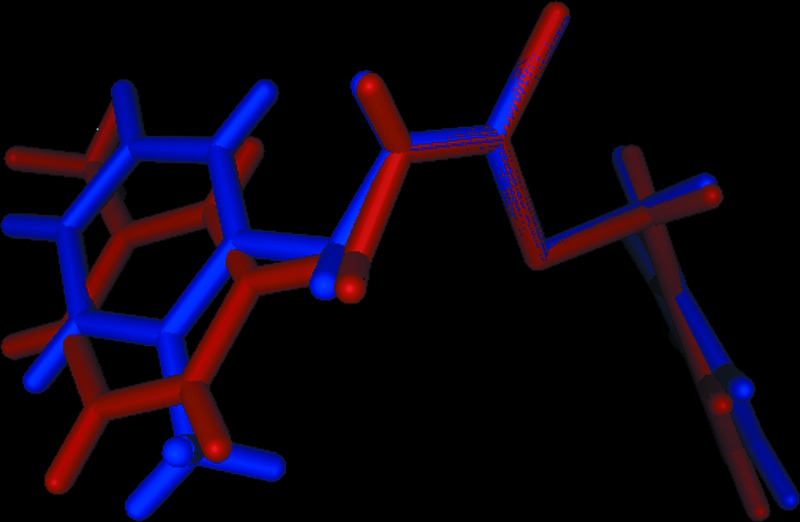
Superimposition of the title compound, shown in red, and the 2-tolyl analogue (blue). The molecules have been superimposed such that the CS2 residues are overlapped.

**Fig. 3.**
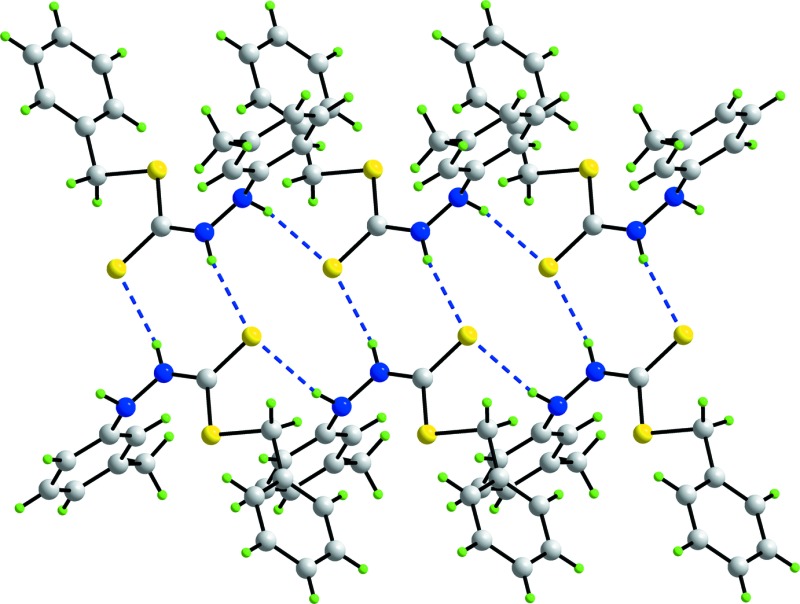
The supramolecular chain along the a axis sustained by N—H···S hydrogen bonding, shown as blue dashed lines.

**Fig. 4.**
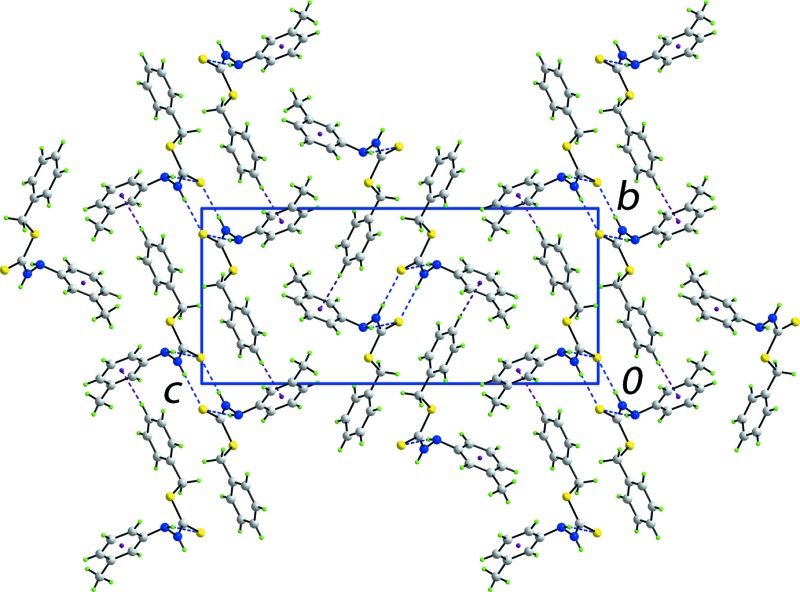
A view of the unit-cell contents in projection down the a axis. The N—H···S and C—H···π interactions are shown as blue and purple dashed lines, respectively.

2 . DOI: 10.1107/S2056989015004764/hb7378fig2.tif
Superimposition of the title compound, shown in red, and the 2-tolyl analogue (blue). The mol­ecules have been superimposed such that the CS_2_ residues are overlapped.

a . DOI: 10.1107/S2056989015004764/hb7378fig3.tif
The supra­molecular chain along the *a* axis sustained by N—H⋯S hydrogen bonding, shown as blue dashed lines.

a . DOI: 10.1107/S2056989015004764/hb7378fig4.tif
A view of the unit-cell contents in projection down the *a* axis. The N—H⋯S and C—H⋯π inter­actions are shown as blue and purple dashed lines, respectively.

CCDC reference: 1052727

Additional supporting information:  crystallographic information; 3D view; checkCIF report

## Figures and Tables

**Table 1:** Hydrogen-bond geometry (, )

*D*H*A*	*D*H	H*A*	*D* *A*	*D*H*A*
N1H1*N*S2^i^	0.88(1)	2.50(2)	3.3581(13)	167(2)
N2H2*N*S2^ii^	0.87(1)	2.52(1)	3.3819(13)	167(2)
C6H6*Cg*1^iii^	0.95	2.61	3.5314(19)	161

Symmetry codes: (i) 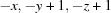; (ii) 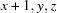; (iii) 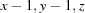.

## supplementary crystallographic information

### S1. Experimental

The compound was prepared according to Tarafder *et al.* (2002). *m*-Tolylhydrazine hydrochloride (0.05 mol) was added to a solution of potassium hydroxide (0.05 mol) in 95% ethanol (70 ml) with continual stirring. This mixture was cooled in an ice-bath until the temperature was about 268 K when the mixture was filtered to remove excess potassium chloride. Carbon disulfide (0.05 mol) was added drop-wise to the filtrate with vigorous stirring for about 1 h. The mixture was kept in the ice-salt bath while benzyl chloride (0.05 mol) was added drop-wise with vigorous stirring. Stirring was continued for 1 h after the complete addition of benzyl chloride. A pale-pink precipitate that was obtained was filtered and washed with cold ethanol. The product was dried in a desiccator and the filtrate was kept in freezer overnight. Pale-brown crystals were obtained from its filtrate. Yield 53.1%. *M*.pt: 416 K. Anal. Found (Calc.): C, 61.26 (62.46); H, 5.42 (5.59); N, 9.02 (9.71)%.

### S2. Refinement

Carbon-bound H-atoms were placed in calculated positions (C—H = 0.95 to 0.99 Å) and were included in the refinement in the riding model approximation with *U_iso_*(H) = 1.2–1.5*U_eq_*(C). The N—H H atoms were refined with N—H = 0.88±0.01 Å, and with *U_iso_*(H) = 1.2*U_eq_*(N). Owing to poor agreement, the (0 0 2) reflection was omitted from the final cycles of refinement.

### Crystal data

**Table:**

C_15_H_16_N_2_S_2_	*F*(000) = 608
*M**_r_* = 288.42	*D*_x_ = 1.337 Mg m^−^^3^
Monoclinic, *P*2_1_/*n*	Cu *K*α radiation, λ = 1.5418 Å
*a* = 5.9396 (1) Å	Cell parameters from 9462 reflections
*b* = 10.3243 (2) Å	θ = 4.7–71.5°
*c* = 23.5474 (5) Å	µ = 3.25 mm^−^^1^
β = 96.952 (2)°	*T* = 100 K
*V* = 1433.36 (5) Å^3^	Prism, pale-brown
*Z* = 4	0.20 × 0.09 × 0.06 mm

### Data collection

**Table:**

Oxford Diffraction Xcaliber Eos Gemini diffractometer	2784 independent reflections
Radiation source: fine-focus sealed tube	2616 reflections with *I* > 2σ(*I*)
Graphite monochromator	*R*_int_ = 0.022
Detector resolution: 16.1952 pixels mm^-1^	θ_max_ = 71.4°, θ_min_ = 4.7°
ω scans	*h* = −7→7
Absorption correction: multi-scan (*CrysAlis PRO*; Agilent, 2011)	*k* = −12→12
*T*_min_ = 0.700, *T*_max_ = 1.000	*l* = −28→28
18486 measured reflections	

### Refinement

**Table:**

Refinement on *F*^2^	2 restraints
Least-squares matrix: full	Hydrogen site location: mixed
*R*[*F*^2^ > 2σ(*F*^2^)] = 0.036	H atoms treated by a mixture of independent and constrained refinement
*wR*(*F*^2^) = 0.104	*w* = 1/[σ^2^(*F*_o_^2^) + (0.0719*P*)^2^ + 0.7171*P*] where *P* = (*F*_o_^2^ + 2*F*_c_^2^)/3
*S* = 1.01	(Δ/σ)_max_ = 0.015
2784 reflections	Δρ_max_ = 0.43 e Å^−^^3^
179 parameters	Δρ_min_ = −0.31 e Å^−^^3^

### Special details

**Table:**

Geometry. All e.s.d.'s (except the e.s.d. in the dihedral angle between two l.s. planes) are estimated using the full covariance matrix. The cell e.s.d.'s are taken into account individually in the estimation of e.s.d.'s in distances, angles and torsion angles; correlations between e.s.d.'s in cell parameters are only used when they are defined by crystal symmetry. An approximate (isotropic) treatment of cell e.s.d.'s is used for estimating e.s.d.'s involving l.s. planes.

### Fractional atomic coordinates and isotropic or equivalent isotropic displacement parameters (Å^2^)

**Table:**

	*x*	*y*	*z*	*U*_iso_*/*U*_eq_	
S1	−0.02626 (6)	0.14613 (3)	0.57928 (2)	0.01811 (14)	
S2	−0.27407 (6)	0.35220 (3)	0.50311 (2)	0.01879 (14)	
N1	0.1325 (2)	0.37365 (13)	0.56063 (5)	0.0185 (3)	
H1N	0.145 (3)	0.4481 (12)	0.5433 (8)	0.022*	
N2	0.3251 (2)	0.32132 (13)	0.59271 (6)	0.0190 (3)	
H2N	0.440 (2)	0.319 (2)	0.5728 (7)	0.023*	
C1	−0.0490 (3)	0.29984 (14)	0.54689 (6)	0.0166 (3)	
C2	−0.2885 (3)	0.06797 (15)	0.54854 (7)	0.0220 (3)	
H2A	−0.4205	0.1231	0.5541	0.026*	
H2B	−0.2867	0.0541	0.5070	0.026*	
C3	−0.3054 (3)	−0.06035 (15)	0.57852 (6)	0.0183 (3)	
C4	−0.4833 (3)	−0.08287 (17)	0.61097 (7)	0.0232 (3)	
H4	−0.5904	−0.0163	0.6152	0.028*	
C5	−0.5047 (3)	−0.20200 (18)	0.63711 (7)	0.0277 (4)	
H5	−0.6282	−0.2171	0.6584	0.033*	
C6	−0.3471 (3)	−0.29890 (17)	0.63237 (7)	0.0289 (4)	
H6	−0.3606	−0.3799	0.6508	0.035*	
C7	−0.1689 (3)	−0.27671 (16)	0.60040 (7)	0.0283 (4)	
H7	−0.0606	−0.3430	0.5968	0.034*	
C8	−0.1488 (3)	−0.15806 (16)	0.57376 (7)	0.0232 (4)	
H8	−0.0263	−0.1437	0.5520	0.028*	
C9	0.3914 (3)	0.37731 (15)	0.64746 (6)	0.0182 (3)	
C10	0.2516 (3)	0.45821 (15)	0.67466 (6)	0.0194 (3)	
H10	0.1050	0.4796	0.6563	0.023*	
C11	0.3250 (3)	0.50851 (16)	0.72891 (7)	0.0231 (3)	
C12	0.5383 (3)	0.47294 (18)	0.75585 (7)	0.0266 (4)	
H12	0.5897	0.5053	0.7929	0.032*	
C13	0.6756 (3)	0.39062 (18)	0.72868 (7)	0.0270 (4)	
H13	0.8197	0.3662	0.7476	0.032*	
C14	0.6050 (3)	0.34342 (16)	0.67428 (7)	0.0220 (3)	
H14	0.7014	0.2886	0.6556	0.026*	
C15	0.1791 (3)	0.60228 (19)	0.75688 (8)	0.0304 (4)	
H15A	0.1134	0.5586	0.7879	0.046*	
H15B	0.0571	0.6339	0.7285	0.046*	
H15C	0.2718	0.6756	0.7724	0.046*	

### Atomic displacement parameters (Å^2^)

**Table:**

	*U*^11^	*U*^22^	*U*^33^	*U*^12^	*U*^13^	*U*^23^
S1	0.0189 (2)	0.0149 (2)	0.0195 (2)	−0.00064 (12)	−0.00198 (15)	0.00260 (13)
S2	0.0174 (2)	0.0191 (2)	0.0192 (2)	0.00001 (13)	−0.00053 (15)	0.00490 (13)
N1	0.0193 (6)	0.0165 (6)	0.0189 (6)	−0.0008 (5)	−0.0012 (5)	0.0036 (5)
N2	0.0166 (6)	0.0212 (7)	0.0189 (6)	0.0010 (5)	0.0009 (5)	0.0006 (5)
C1	0.0204 (7)	0.0157 (7)	0.0141 (6)	0.0019 (6)	0.0037 (5)	−0.0002 (5)
C2	0.0194 (7)	0.0187 (8)	0.0259 (8)	−0.0037 (6)	−0.0055 (6)	0.0019 (6)
C3	0.0202 (7)	0.0177 (7)	0.0160 (7)	−0.0026 (6)	−0.0020 (6)	−0.0007 (6)
C4	0.0188 (7)	0.0288 (9)	0.0214 (7)	0.0000 (6)	−0.0001 (6)	−0.0016 (7)
C5	0.0227 (8)	0.0404 (10)	0.0197 (8)	−0.0116 (7)	0.0013 (6)	0.0046 (7)
C6	0.0350 (9)	0.0233 (9)	0.0265 (8)	−0.0089 (7)	−0.0046 (7)	0.0073 (7)
C7	0.0344 (9)	0.0183 (8)	0.0316 (9)	0.0033 (7)	0.0019 (7)	0.0023 (7)
C8	0.0256 (8)	0.0218 (8)	0.0228 (8)	0.0002 (6)	0.0055 (6)	0.0010 (6)
C9	0.0191 (7)	0.0178 (7)	0.0174 (7)	−0.0044 (6)	0.0009 (6)	0.0038 (6)
C10	0.0180 (7)	0.0192 (7)	0.0206 (7)	−0.0017 (6)	0.0012 (6)	0.0040 (6)
C11	0.0266 (8)	0.0224 (8)	0.0211 (7)	−0.0035 (6)	0.0063 (6)	0.0019 (6)
C12	0.0288 (8)	0.0333 (9)	0.0169 (7)	−0.0065 (7)	−0.0006 (6)	0.0005 (7)
C13	0.0217 (8)	0.0344 (9)	0.0233 (8)	−0.0016 (7)	−0.0036 (6)	0.0057 (7)
C14	0.0194 (8)	0.0235 (8)	0.0230 (8)	0.0006 (6)	0.0021 (6)	0.0041 (6)
C15	0.0353 (9)	0.0319 (10)	0.0248 (8)	−0.0013 (8)	0.0070 (7)	−0.0059 (7)

### Geometric parameters (Å, º)

**Table:**

S1—C1	1.7588 (15)	C6—H6	0.9500
S1—C2	1.8245 (16)	C7—C8	1.388 (2)
S2—C1	1.6761 (15)	C7—H7	0.9500
N1—C1	1.328 (2)	C8—H8	0.9500
N1—N2	1.4007 (18)	C9—C10	1.388 (2)
N1—H1N	0.878 (9)	C9—C14	1.392 (2)
N2—C9	1.424 (2)	C10—C11	1.399 (2)
N2—H2N	0.875 (9)	C10—H10	0.9500
C2—C3	1.510 (2)	C11—C12	1.396 (2)
C2—H2A	0.9900	C11—C15	1.503 (2)
C2—H2B	0.9900	C12—C13	1.386 (3)
C3—C8	1.386 (2)	C12—H12	0.9500
C3—C4	1.397 (2)	C13—C14	1.387 (2)
C4—C5	1.388 (3)	C13—H13	0.9500
C4—H4	0.9500	C14—H14	0.9500
C5—C6	1.384 (3)	C15—H15A	0.9800
C5—H5	0.9500	C15—H15B	0.9800
C6—C7	1.390 (3)	C15—H15C	0.9800
			
C1—S1—C2	102.12 (7)	C8—C7—H7	119.9
C1—N1—N2	119.73 (13)	C6—C7—H7	119.9
C1—N1—H1N	120.1 (13)	C3—C8—C7	120.68 (15)
N2—N1—H1N	118.6 (13)	C3—C8—H8	119.7
N1—N2—C9	116.74 (13)	C7—C8—H8	119.7
N1—N2—H2N	111.2 (13)	C10—C9—C14	120.35 (15)
C9—N2—H2N	110.3 (13)	C10—C9—N2	123.14 (14)
N1—C1—S2	121.90 (11)	C14—C9—N2	116.47 (14)
N1—C1—S1	113.26 (11)	C9—C10—C11	120.48 (14)
S2—C1—S1	124.84 (9)	C9—C10—H10	119.8
C3—C2—S1	107.71 (10)	C11—C10—H10	119.8
C3—C2—H2A	110.2	C12—C11—C10	118.85 (15)
S1—C2—H2A	110.2	C12—C11—C15	120.66 (15)
C3—C2—H2B	110.2	C10—C11—C15	120.46 (15)
S1—C2—H2B	110.2	C13—C12—C11	120.25 (15)
H2A—C2—H2B	108.5	C13—C12—H12	119.9
C8—C3—C4	118.88 (15)	C11—C12—H12	119.9
C8—C3—C2	121.17 (14)	C12—C13—C14	120.85 (15)
C4—C3—C2	119.94 (14)	C12—C13—H13	119.6
C5—C4—C3	120.39 (16)	C14—C13—H13	119.6
C5—C4—H4	119.8	C13—C14—C9	119.18 (16)
C3—C4—H4	119.8	C13—C14—H14	120.4
C6—C5—C4	120.40 (15)	C9—C14—H14	120.4
C6—C5—H5	119.8	C11—C15—H15A	109.5
C4—C5—H5	119.8	C11—C15—H15B	109.5
C5—C6—C7	119.42 (15)	H15A—C15—H15B	109.5
C5—C6—H6	120.3	C11—C15—H15C	109.5
C7—C6—H6	120.3	H15A—C15—H15C	109.5
C8—C7—C6	120.22 (16)	H15B—C15—H15C	109.5
			
C1—N1—N2—C9	−117.48 (15)	C2—C3—C8—C7	−178.47 (15)
N2—N1—C1—S2	−173.66 (10)	C6—C7—C8—C3	−0.1 (3)
N2—N1—C1—S1	6.62 (17)	N1—N2—C9—C10	14.4 (2)
C2—S1—C1—N1	−176.50 (11)	N1—N2—C9—C14	−167.76 (13)
C2—S1—C1—S2	3.79 (12)	C14—C9—C10—C11	1.1 (2)
C1—S1—C2—C3	−173.00 (11)	N2—C9—C10—C11	178.89 (14)
S1—C2—C3—C8	−64.36 (17)	C9—C10—C11—C12	−1.8 (2)
S1—C2—C3—C4	116.71 (14)	C9—C10—C11—C15	176.39 (15)
C8—C3—C4—C5	−1.1 (2)	C10—C11—C12—C13	0.9 (2)
C2—C3—C4—C5	177.82 (14)	C15—C11—C12—C13	−177.29 (16)
C3—C4—C5—C6	1.4 (2)	C11—C12—C13—C14	0.7 (3)
C4—C5—C6—C7	−1.0 (3)	C12—C13—C14—C9	−1.4 (3)
C5—C6—C7—C8	0.4 (3)	C10—C9—C14—C13	0.5 (2)
C4—C3—C8—C7	0.5 (2)	N2—C9—C14—C13	−177.39 (14)

### Hydrogen-bond geometry (Å, º)

**Table:**

*D*—H···*A*	*D*—H	H···*A*	*D*···*A*	*D*—H···*A*
N1—H1*N*···S2^i^	0.88 (1)	2.50 (2)	3.3581 (13)	167 (2)
N2—H2*N*···S2^ii^	0.87 (1)	2.52 (1)	3.3819 (13)	167 (2)
C6—H6···*Cg*1^iii^	0.95	2.61	3.5314 (19)	161

Symmetry codes: (i) −*x*, −*y*+1, −*z*+1; (ii) *x*+1, *y*, *z*; (iii) *x*−1, *y*−1, *z*.
