# Supplementary figures and images for: Crystal structure of benzyl 3-(3-methyl­phen­yl)di­thio­carbazate
Source: Acta Crystallogr E Crystallogr Commun. 2015 Mar 14;71(Pt 4):o233–4. doi: 10.1107/S2056989015004764 (PMC4438800; doi:10.1107/S2056989015004764)

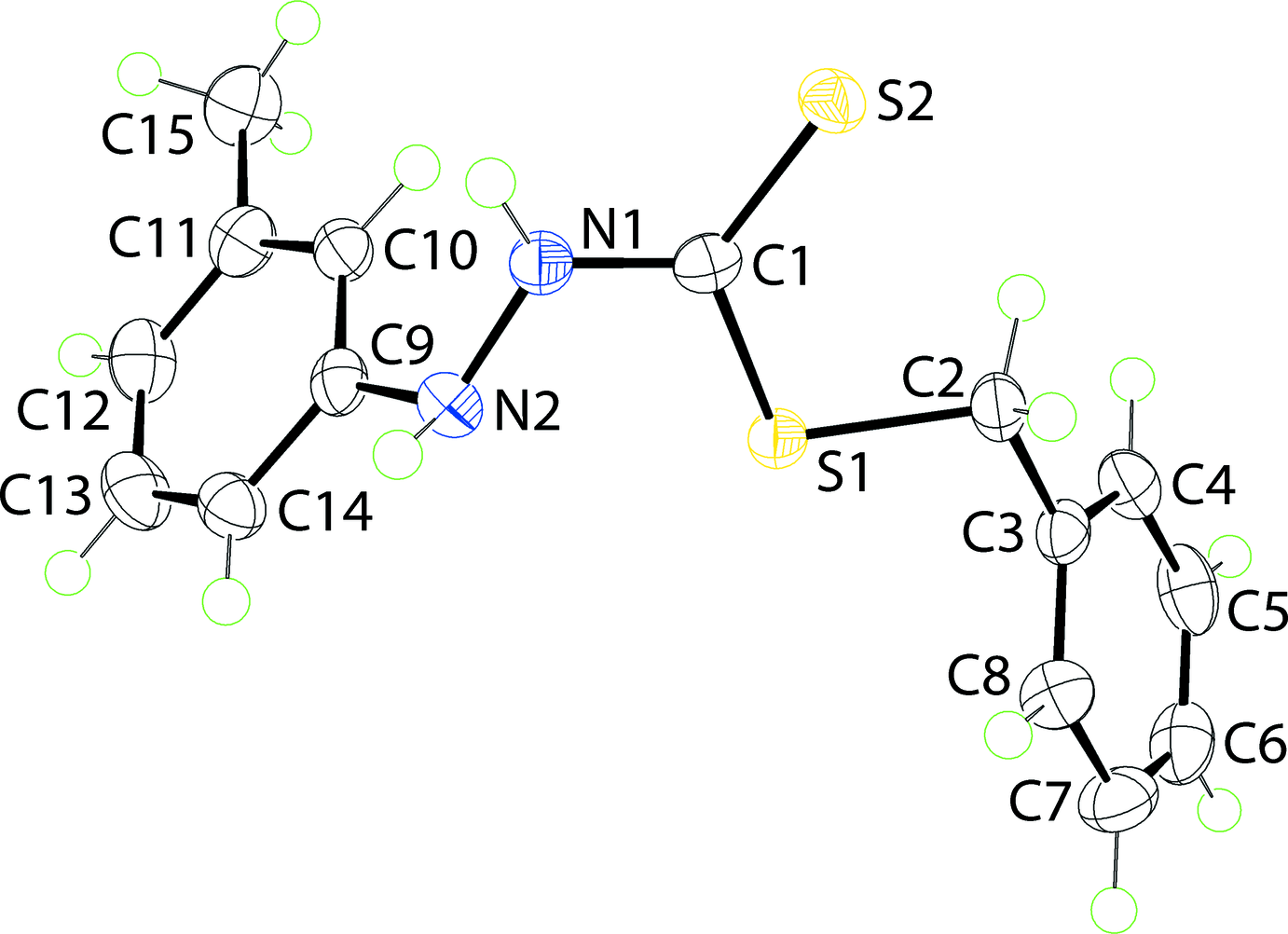

Supplement: Supplementary file 4 [file e-71-0o233-fig1.tif]

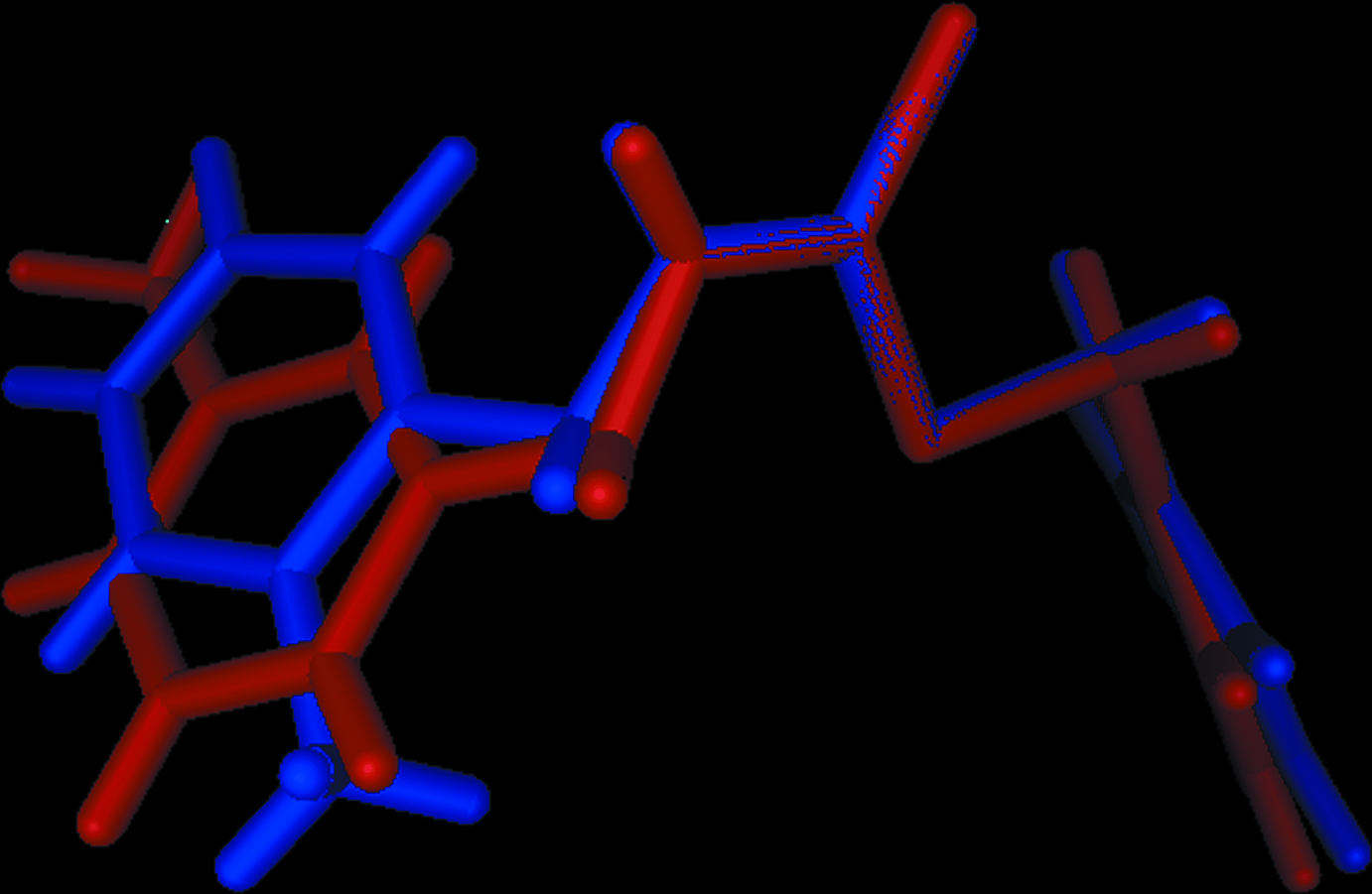

Supplement: Supplementary file 5 [file e-71-0o233-fig2.tif]

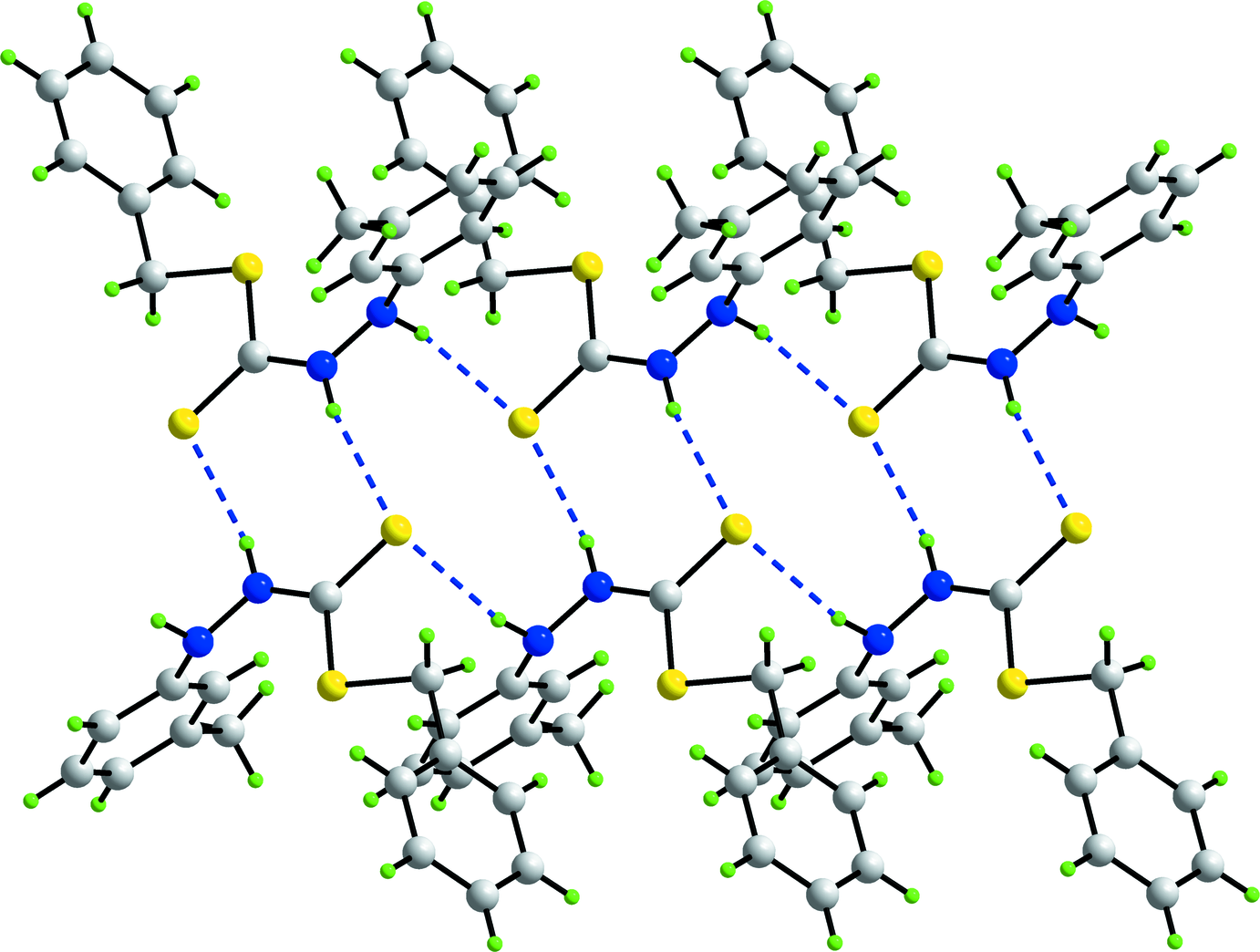

Supplement: Supplementary file 6 [file e-71-0o233-fig3.tif]

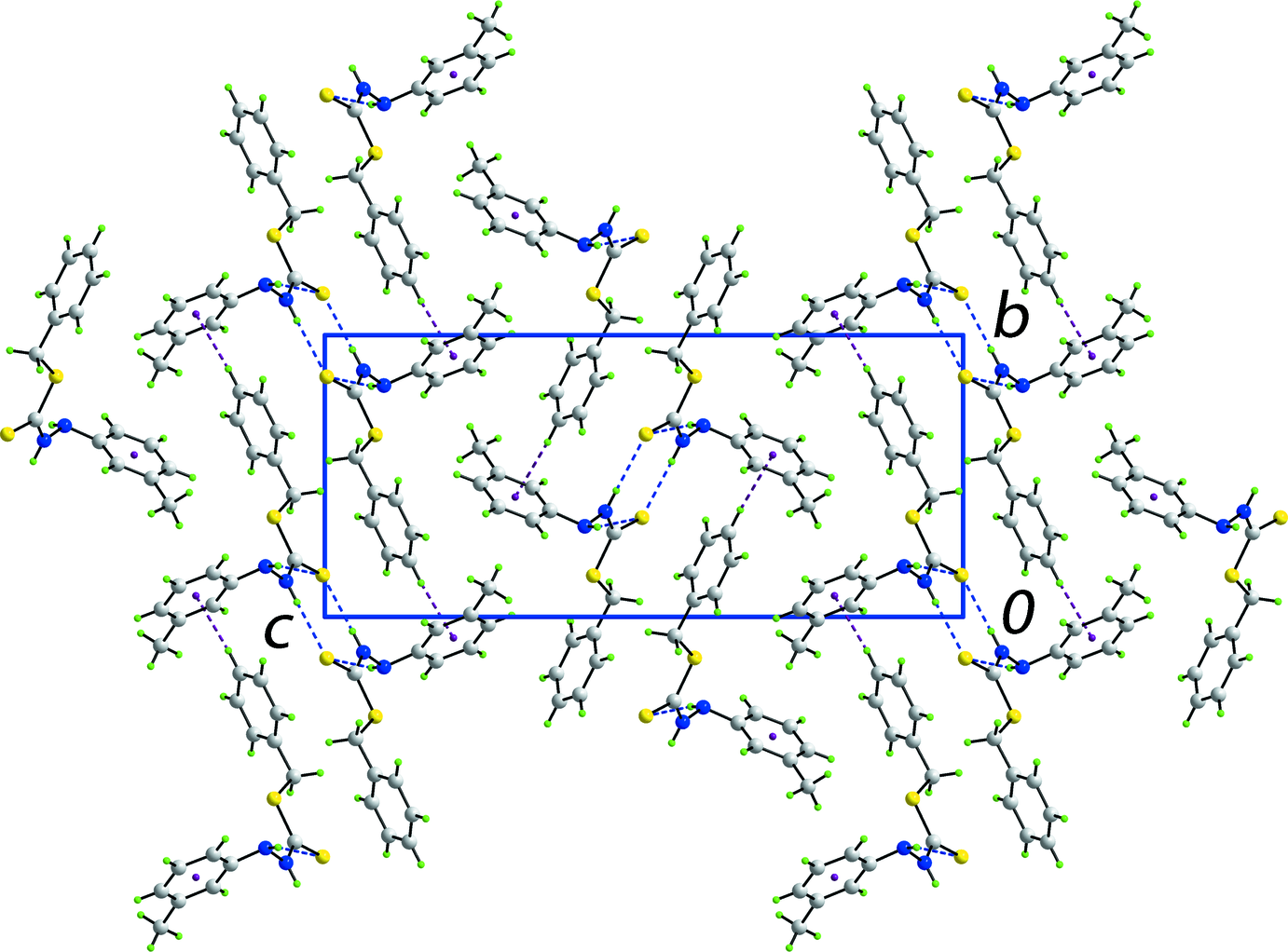

Supplement: Supplementary file 7 [file e-71-0o233-fig4.tif]
